# Supplementary material for: Self-Management of Medication on a Cardiology Ward: Feasibility and Safety of the SelfMED Intervention
Source: Int J Environ Res Public Health. 2022 Dec 13;19(24):16715. doi: 10.3390/ijerph192416715 (PMC9778667; doi:10.3390/ijerph192416715)
Supplement: Supplementary file 1 [file ijerph-19-16715-s001.zip › Supplementary File S2.pdf]

Post-intervention survey "Patient self-management of medication"

Dear healthcare provider,

The intervention study on "Patient self-management of medication" in the cardiology department is coming to an end. Since you participated in this study, we would like to ask you some questions about how you experienced the intervention. For each question, please tick off or circle the most appropriate answer.

Thank you very much for your participation.

1. What is your profession? Tick off.
   - o Physician
   - o Nurse
   - o Hospital pharmacist
   - o Pharmacy assistant
   - o Other: ………………………………………………………….

2. On a scale of 1 to 10, please indicate how satisfied you are with the requested item.

2.1. The overall intervention 'self-management of medication by patients’.

1 2 3 4 5 6 7 8 9 10

Absolutely not satisfied — Absolutely satisfied

2.2. The items surveyed in the nurse assessment.

1 2 3 4 5 6 7 8 9 10

Absolutely not satisfied — Absolutely satisfied

2.3. The items surveyed in the patient self-assessment.

1 2 3 4 5 6 7 8 9 10

Absolutely not satisfied — Absolutely satisfied

2.4. The number of items surveyed in the nurse assessment.

1 2 3 4 5 6 7 8 9 10

Absolutely not satisfied — Absolutely satisfied

2.5. Het number of items surveyed in the patient self-assessment.

1 2 3 4 5 6 7 8 9 10

Absolutely not satisfied — Absolutely satisfied

2.6. The user-friendliness of the script.

1 2 3 4 5 6 7 8 9 10

Absolutely not satisfied — Absolutely satisfied

2.7. The time investment for enabling patient self-management of medication.

1 2 3 4 5 6 7 8 9 10

Absolutely not satisfied — Absolutely satisfied

3. On a scale of 1 to 10, please indicate the extent to which you agree with the statement.

3.1. The intervention contributes to improved patient self-management in terms of the medication therapy to be followed.

1	2	3	4	5	6	7	8	9	10
Strongly disagree									Strongly agree

3.2. The intervention contributes to improved communication between patients, nurses and physicians regarding medication therapy.

1	2	3	4	5	6	7	8	9	10
Strongly disagree									Strongly agree

3.3. The intervention contributes to a better understanding of the patient's competences to continue taking medication correctly at home after discharge.

1	2	3	4	5	6	7	8	9	10
Strongly disagree									Strongly agree

4. Do you have any other comments? You can note them below.

………
………
………
